# Enigmatic persistence of aerobic methanotrophs in oxygen-limiting freshwater habitats

**DOI:** 10.1093/ismejo/wrae041

**Published:** 2024-03-12

**Authors:** Paula C J Reis, Jackson M Tsuji, Cerrise Weiblen, Sherry L Schiff, Matthew Scott, Lisa Y Stein, Josh D Neufeld

**Affiliations:** Department of Biology, University of Waterloo, Waterloo, ON N2L 3G1, Canada; Super-cutting-edge Grand and Advanced Research (SUGAR) Program, Institute for Extra-cutting-edge Science and Technology Avant-garde Research, Japan Agency for Marine-Earth Science and Technology, Yokosuka, Kanagawa, Japan; Department of Biological Sciences, University of Alberta, Edmonton, AB T6G 2E9, Canada; Department of Earth & Environmental Sciences, University of Waterloo, Waterloo, ON N2L 3G1, Canada; Department of Biology, University of Waterloo, Waterloo, ON N2L 3G1, Canada; Department of Biological Sciences, University of Alberta, Edmonton, AB T6G 2E9, Canada; Department of Biology, University of Waterloo, Waterloo, ON N2L 3G1, Canada

**Keywords:** methane-oxidizing bacteria, methane oxidation, anoxia, electron acceptor, freshwater, aquatic ecosystem

## Abstract

Methanotrophic bacteria mitigate emissions of the potent greenhouse gas methane (CH_4_) from a variety of anthropogenic and natural sources, including freshwater lakes, which are large sources of CH_4_ on a global scale. Despite a dependence on dioxygen (O_2_) for CH_4_ oxidation, abundant populations of putatively aerobic methanotrophs have been detected within microoxic and anoxic waters and sediments of lakes. Experimental work has demonstrated active aerobic methanotrophs under those conditions, but how they are able to persist and oxidize CH_4_ under O_2_ deficiency remains enigmatic. In this review, we discuss possible mechanisms that underpin the persistence and activity of aerobic methanotrophs under O_2_-limiting conditions in freshwater habitats, particularly lakes, summarize experimental evidence for microbial oxidation of CH_4_ by aerobic bacteria under low or no O_2_, and suggest future research directions to further explore the ecology and metabolism of aerobic methanotrophs in O_2_-limiting environments.

## Introduction

Using methane (CH_4_) as a source of carbon and energy, methanotrophs serve as a biological sink for this potent greenhouse gas in terrestrial, freshwater, and marine ecosystems, with impacts on the global CH_4_ cycle [1]. Aquatic ecosystems (freshwater and marine) account for roughly half of the global CH_4_ emissions from natural and anthropogenic sources [2]. Despite the relatively small global area covered by freshwaters (about 3% of Earth’s land surface [3]), these ecosystems are critical sources of CH_4_ to the atmosphere, emitting roughly 159 Tg of CH_4_ year^−1^, which is equivalent to 25% of the land-associated greenhouse gas sink [4, 5].

In lakes, methanotrophic bacteria [or methane-oxidizing bacteria (MOB)] are particularly relevant for controlling diffusive CH_4_ emissions to the atmosphere. In stratified water columns, they often consume all upward diffusive flux of sedimentary CH_4_ and thereby prevent much greater emissions [6-8]. Despite this importance, uncertainty remains regarding microbial strategies for CH_4_ oxidation in lakes, especially those related to the enigmatic oxidation of CH_4_ by aerobic methanotrophic bacteria in microoxic and anoxic lake waters and sediments. Such oxidation of CH_4_ under O_2_-deficient conditions in lakes could be significant and has been estimated to consume roughly one-third of the total CH_4_ produced in deep lake sediments [9]. As human activities lead to future deoxygenation of lake water columns [10-12] and increased lake CH_4_ production and emissions [13, 14], understanding mechanisms for microbial consumption of this potent greenhouse gas under O_2_-limiting conditions is particularly urgent.

**Figure 1:**
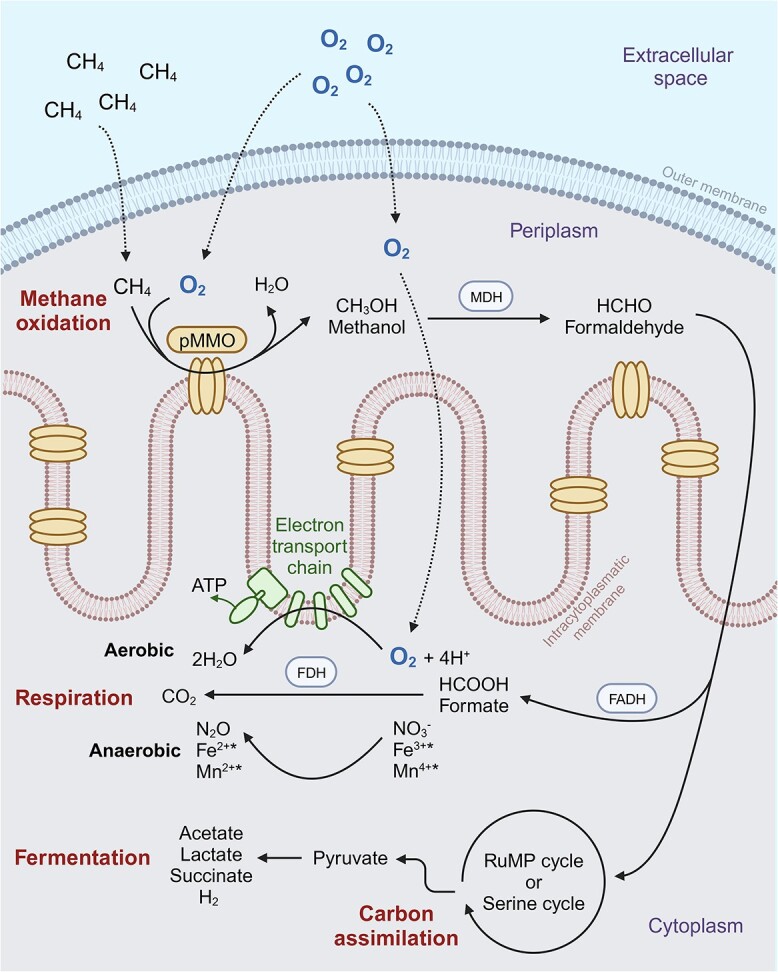
Schematic representation of the possible steps in the metabolism of aerobic methanotrophic bacteria. O_2_ is used in the oxidation of CH_4_ to methanol in the first step catalyzed by MMO enzymes (represented by the pMMO in this scheme). This first step may also be performed in the cytoplasm by the sMMO in methanotrophic bacteria possessing this enzyme. Methanol is converted to formaldehyde by methanol dehydrogenase (MDH); formaldehyde can be assimilated into cellular compounds (via the ribulose monophosphate (RuMP) cycle in gammaproteobacterial MOB or via the serine cycle in alphaproteobacterial MOB) or be oxidized to formate by formaldehyde dehydrogenases (FADH); formate is then oxidized to CO_2_ by formate dehydrogenases (FDH). O_2_ is the terminal electron acceptor in aerobic respiration, while other oxidants can potentially serve as terminal electron acceptors in methanotrophic bacteria performing anaerobic respiration under O_2_-limiting conditions. Under O_2_ limitation, carbon that enters the RuMP cycle can go through fermentation for energy generation as opposed to respiration. ^*^Iron (Fe^3+^) and manganese (Mn^4+^) reduction would presumably occur outside the cell, via extracellular electron transfer, and could potentially be mediated by electron shuttles or microbial consortia. This figure was created with BioRender.

Although considered obligately aerobic (i.e. organisms that survive and grow only in the presence of O_2_), methanotrophic bacteria, particularly members of the class *Gammaproteobacteria*, order *Methylococcales*, appear to be active under seemingly anoxic conditions (i.e. O_2_ levels below sensor detection limit) in stratified lake water columns and sediments (e.g. [15-18]). Aerobic microorganisms can have metabolic versatility that allows their survival under variable O_2_ conditions, such as wide range of O_2_ affinities and ability to use alternative electron acceptors or perform fermentation [19-21]. Aimed at exploring mechanisms for the presence and activity of aerobic methanotrophic bacteria under O_2_-limiting conditions, studies have evaluated the use of alternative electron acceptors such as sulfate, nitrate, nitrite, and iron and manganese oxides coupled to CH_4_ oxidation (e.g. [22-24]) or the potential for cryptic O_2_ cycling to support aerobic methanotrophy (e.g. [15]). Results from such experiments offer evidence for increased CH_4_ oxidation rate with the addition of alternative electron acceptors (varying between studies) or when coupled to the activity of oxygenic photosynthetic microorganisms under illuminated conditions. Therefore, findings are still unclear regarding the role of O_2_ or other electron acceptors in sustaining a presumed O_2_-dependent process within anoxic habitats. Because the enzyme responsible for the first step of CH_4_ oxidation in aerobic MOB [i.e. methane monooxygenase (MMO)] requires O_2_ to activate CH_4_ for its oxidation to methanol (Fig. 1), a completely anaerobic metabolism for these microorganisms appears unlikely.

Studies investigating the genetic makeup of aerobic methanotrophic bacteria in lakes have identified that the genomes of gammaproteobacterial methanotrophs encode genes that confer physiological advantages for survival under O_2_-limited conditions. Such genes encode for denitrification [24], fermentation pathways [25], high affinity oxidases [26], or O_2_ carriers such as bacteriohemerythrins [27, 28]. Therefore, gammaproteobacterial methanotrophs appear to possess some genomic potential to persist under O_2_-depleted conditions, conferring an ability to occupy niches where O_2_ starvation is preferred over CH_4_ starvation [29-31].

Here we review recent research exploring microbial oxidation of CH_4_ by putatively aerobic bacteria under anoxia in limnetic systems. We summarize findings of recently published experimental work and explore the genetic makeup of aerobic methanotrophic bacteria inhabiting O_2_-deprived niches that may permit their growth and activity under such conditions. We also highlight future research directions that may help disentangle mechanisms and ecological strategies of aerobic methanotrophs found within anoxic freshwater environments.

### Anaerobic and aerobic methanotrophs

Methanotrophs use CH_4_ as a source of energy and/or carbon, and they rely on O_2_ or other electron acceptors (e.g. sulfate, SO_4_^2−^; manganese, Mn^4+^; iron, Fe^3+^; nitrate, NO_3_^−^; nitrite, NO_2_^−^) to perform aerobic or anaerobic CH_4_ oxidation, respectively. Known anaerobic methanotrophs (ANME) are archaea in the *Euryarchaeota* phylum belonging to three clades (i.e. ANME-1, 2, and 3) that are affiliated with the orders *Methanophagales* and *Methanosarcinales* [32] (Table S1). All known archaeal methanotrophs are anaerobic and oxidize CH_4_ by performing “reverse” methanogenesis in consortia with sulfate-reducing members of the *Deltaproteobacteria*, or using NO_3_^−^, Fe^3+^, or Mn^4+^ as electron acceptors without a bacterial partner in the case of ANME-2d [33-35]. Methanotrophic archaea are identified by the *mcrA* gene, encoding the alpha subunit of methyl-coenzyme M reductase (MCR), which catalyzes an essential step for both anaerobic methanotrophy and methanogenesis [36]. Methanotrophic archaea are well recognized for their role in reducing atmospheric CH_4_ flux from the ocean floor, where relatively high SO_4_^2−^ concentrations support anaerobic CH_4_ oxidation [36]. ANME representatives have been detected in freshwater [37-41], although to a much lesser extent. Lower SO_4_^2−^ concentrations in freshwater, compared with marine environments, is thought to limit ANME archaea, although *Methanoperedens* ANME have been detected in freshwater wetlands and lake sediments, potentially using electron acceptors other than SO_4_^2−^ (e.g. Fe^3+^ and Mn^4+^) to oxidize CH_4_ or using SO_4_^2−^ made available by cryptic sulfur cycling [42, 43].

**Figure 2:**
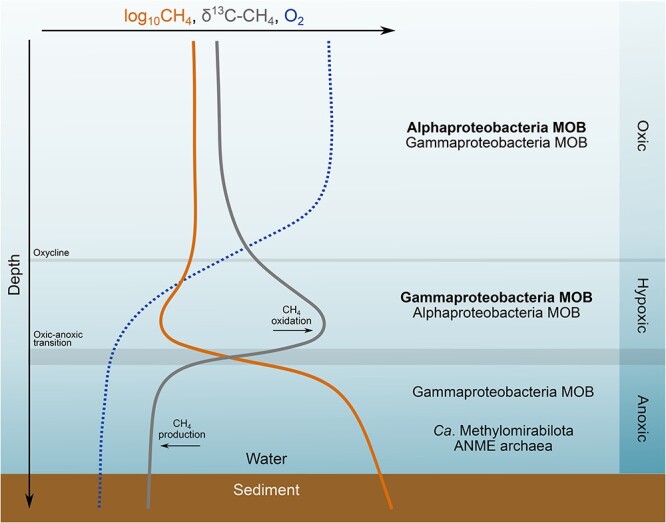
Schematic representation of methane (CH_4_) oxidation zones in seasonally or permanently stratified lakes with anoxic hypolimnia and sediments. Shown are typical profiles of dissolved oxygen (O_2_), CH_4_, and carbon stable isotope composition of CH_4_ and the methanotrophic microorganisms commonly found in each layer. In the oxic and hypoxic portions of the water column, alphaproteobacterial and gammaproteobacterial MOB can be found, with gammaproteobacterial MOB often more abundant in hypoxic waters, as indicated by bold fonts. Gammaproteobacterial MOB, “Ca. Methylomirabilota”, and anaerobic methanotrophic archaea (ANME) can be present in anoxic hypolimnia and sediments.The abundance, activity, and importance of each methanotrophic group to the total CH_4_ consumption in each of the layers may vary with the ecosystem. Methanotrophic bacteria in the “Ca. Methylomirabilota” (formerly NC10) phylum aerobically oxidize CH_4_ under anoxic conditions through intracellular O_2_ production from nitrite.

In contrast to their anaerobic counterparts, all known aerobic methanotrophs are bacteria (Table S1). MOB (or methanotrophs) are a subset of methylotrophs (i.e. aerobic bacteria that use single carbon compounds as carbon and energy sources) [44] and are responsible for oxidizing the sediment CH_4_ flux reaching the oxic-anoxic boundary layer in freshwater lakes and reservoirs [6-8]. The presence of MMO enzymes such as particulate MMO (pMMO) encoded by *pmoCAB* (or *pxmABC* [45]) genes is a defining characteristic of MOB and confers their ability to oxidize CH_4_ requiring one oxygen atom to form to a methanol molecule (Fig. 1). Known MOB comprise a polyphyletic group of bacteria distributed in the *Proteobacteria*, *Verrucomicrobia*, and “*Candidatus* Methylomirabilota” (also known as NC10) phyla [44, 46, 47] (Table S1). MOB within the “Ca. Methylomirabilota” phylum (e.g. “*Ca*. Methylomirabilis oxyfera”) produce O_2_ internally from NO_2_^−^ to perform aerobic CH_4_ oxidation under anoxic conditions [47]. Due to this unique metabolism of “*Ca*. M. oxyfera” and related taxa, these bacteria are referred to as “intra-aerobic” or “anaerobic” methanotrophs in the literature, even though CH_4_ is oxidized via MMO, using O_2_ for activation. MOB in the *Verrucomicrobia* phyla are aerobic methanotrophs typically found in high temperature and low pH environments such as volcanic soils and hot springs [48]. Nearly all MOB possess the copper-containing membrane-bound pMMO, except for facultative methanotrophs in the *Methylocella* genus [49], while an iron-containing soluble MMO (sMMO) is widespread among alphaproteobacterial MOB but is limited among gammaproteobacterial MOB [50, 51]. Therefore, the *pmoA* gene encoding the alpha subunit of pMMO is commonly used as a functional gene marker of aerobic methanotrophic bacteria in the environment. The origin and proliferation of methanotrophy in different bacterial clades remains unclear. Within the *Proteobacteria*, some evidence suggests vertical descent based on phylogenetic congruence between 16S rRNA and *pmoA* sequence analyses [52], whereas other pieces of evidence point to horizontal gene transfer [53, 54]. Despite their aerobic nature, proteobacterial methanotrophs, particularly members of the class *Gammaproteobacteria*, order *Methylococcales*, have been detected under a wide range of O_2_ concentrations in freshwater environments, including under anoxia (e.g. [16, 17, 30, 55, 56]; Fig. 2).

### Zones of methane oxidation in stratified lakes

For stratified lake water columns, a typical CH_4_ profile shows minimum concentrations near the oxic-anoxic transition zone in the water column, concomitant with maximum values for CH_4_ stable carbon isotopic values (Fig. 2; e.g. [6, 39, 57-60]) and highest aerobic MOB abundances (e.g. [57, 61]). The slower reaction of the heavier stable carbon isotope (^13^C) during aerobic CH_4_ oxidation results in the high carbon stable isotopic values of CH_4_ (i.e. isotopic fractionation) in this zone (Fig. 2; [62]). This pattern indicates that aerobic methanotrophs are maximally active in this transition zone where both key substrates for their metabolism (i.e. CH_4_ and O_2_) are available. Typical carbon stable isotopic values of CH_4_ (δ^13^C-CH_4_) in water column oxic-anoxic transition zones range between −50‰ and − 15‰ [58], but could be as high as 0‰ [57], or potentially positive. In temperate lakes, methane oxidation rates at this maximum activity zone can vary widely depending on the lake (0.1–100 μmol CH_4_ L^−1^ d^−1^) [18]. Oxic-anoxic interfaces with high aerobic methanotrophic activity and enriched δ^13^C-CH_4_ can also be situated in littoral sediment, in deeper sediments close to roots releasing O_2_, or in the surface sediment in unstratified water columns. The zone with maximal methanotrophic activity is usually confined to a layer of only a few millimeters in the sediment but can be extended over the meter scale in the water column (Fig. 2) and follows the movements of the groundwater table in wetlands [8].

In overlying oxygenated mixed layers, aerobic methanotrophic bacteria can also oxidize CH_4_ that may have escaped oxidation at the oxic-anoxic transition zone, as well as CH_4_ produced in anoxic littoral sediments or within the oxygenated water column [63], although at rates typically one or two orders of magnitude lower than that at the oxic-anoxic transition zone (0.01–0.1 μmol CH_4_ L^−1^ d^−1^) [18]. δ^13^C-CH_4_ values in the oxic surface layers reflect the lower CH_4_ oxidation rates and other CH_4_ sources (littoral sediments, oxic CH_4_ production), being typically more negative than δ^13^C-CH_4_ values at the oxic-anoxic zone but more positive than in the deep anoxic layer (Fig. 2). Vertical stratification patterns of methanotrophs have been observed in lake water columns, with alphaproteobacterial methanotrophs being more abundant in well-oxygenated upper layers, and gammaproteobacterial methanotrophs more abundant at greater depths under lower O_2_ concentrations [18, 40, 64] (Fig. 2).

Below the oxic-anoxic transition zone of permanently or temporarily stratified lakes, discernable stable isotope enrichment in CH_4_ is often not observed (Fig. 2; e.g. [23, 30, 57, 60, 65]), although one previous study reported ^13^CH_4_ enrichment in anoxic waters of a stratified boreal lake [17]. Despite no evident change in stable isotopes of CH_4_, the presence of “Ca. Methylomirabilota” bacteria, ANME archaea, and aerobic methanotrophs of the class *Gammaproteobacteria* (order *Methylococcales*) in anoxic water or sediments of lakes (Fig. 2; e.g. [16, 17, 30, 55, 56, 66]) suggests that CH_4_ oxidation may also occur at those depths. For aerobic methanotrophs (MOB), activity-based evidence such as *in vitro* methane oxidation rate measurements combined with stable isotope probing techniques where ANME archaea and “Ca. Methylomirabilota” are absent but MOB are present (e.g. [9, 15, 67, 68]) has supported this hypothesis, but the mechanisms allowing such activity in the absence of O_2_ remain unclear. Higher rates and stronger isotopic fractionation of methanogenesis than those of methanotrophy likely mask stable isotopic signatures of methanotrophic activity under anoxia in lake profiles, leading to a “silent” but potentially important anaerobic CH_4_ filter [9, 23]. ^13^C-depleted particulate organic matter in the anoxic hypolimnia of lakes also suggests microbial CH_4_ oxidation with assimilation of CH_4_-derived carbon into microbial biomass in the absence of O_2_ [66]. In addition, diffusion from a sedimentary source alone (i.e. solely due to concentration gradients) cannot always fully account for observed CH_4_ concentration profiles in anoxic hypolimnia [55], which implies the presence of a CH_4_ oxidation pathway that explains lower CH_4_ concentrations under these conditions.

### Methane oxidation by aerobic bacteria in O_2_-limiting lake waters and sediments

**Figure 3:**
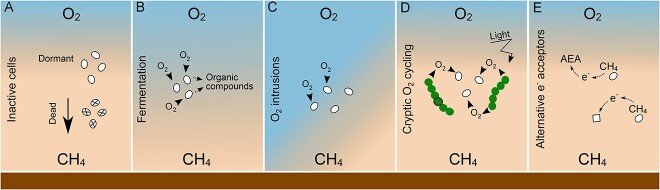
Graphical depiction of hypotheses for the presence of MOB (ovals) in microoxic or anoxic waters of stratified lakes. The panels show the counter gradient of O_2_ (top) and CH_4_ (bottom) in water columns and the sediment (horizontal bar below). a. under anoxic conditions, MOB cells could be inactive, either dormant or dead; b. under microoxic conditions, MOB may aerobically oxidize CH_4_ and perform fermentation with the release of CH_4_-derived organic compounds and low biomass accumulation; c. downward O_2_ diffusive flux or episodic O_2_ intrusions due to water column instability could supply O_2_ to MOB to oxidize CH_4_ aerobically at depths that appear anoxic at the time of sampling; d. O_2_ supply via local photosynthetic O_2_ production could sustain aerobic CH_4_ oxidation by MOB in seemingly anoxic waters (filaments depict oxygenic photosynthetic microorganisms); e. MOB could potentially use alternative electron acceptors (AEA) to oxidize CH_4_ and/or perform anaerobic respiration under anoxic conditions, which could involve the participation of other microorganisms (squares) in syntrophic relationships with MOB.

The occurrence of aerobic MOB in O_2_-limited layers of freshwater environments remains enigmatic, and several reasons could explain their presence under microoxic or anoxic conditions (Fig. 3). First, as the presence of DNA does not necessarily imply active or viable cells, MOB detected in O_2_-deprived environments could be dormant [69] or dead [56] with no consequence to CH_4_ cycling (Fig. 3a). Cells may turn dormant when O_2_ becomes limiting and stay inactive (not oxidizing CH_4_) until conditions become favorable or die and sink into deeper waters. Differently, by coupling CH_4_ oxidation to fermentation or anaerobic respiration using nitrate or other electron acceptors (Fig. 1), bacterial methanotrophs are thought to reduce the O_2_ requirement for respiration, allowing higher O_2_ availability for CH_4_ oxidation. Fermentation is demonstrated in hypoxic cultures where gammaproteobacterial MOB exhibit low biomass accumulation and release of CH_4_-derived organic compounds [25, 29] (Fig. 3b). In low O_2_ conditions, a fermentation mode with high excretion of CH_4_-derived organic compounds has been hypothesized to enable MOB survival and sustaining of other microbial populations in lake hypolimnia [65, 70, 71]. Culture work has also demonstrated coupling of nitrate respiration to CH_4_ oxidation in a gammaproteobacterial MOB representative [24] (Fig. 1). However, to oxidize CH_4_, these microorganisms must use O_2_ or another electron acceptor. Regarding O_2_, downward O_2_ diffusive flux or episodic O_2_ supply [30, 72], and cryptic O_2_ cycling with local photosynthetic O_2_ production [15], have been hypothesized to sustain aerobic CH_4_ oxidation in seemingly anoxic habitats in lakes (Fig. 3c and d). Alternatively, the use of an alternative electron acceptor potentially directly involved in the activation of CH_4_, as well as syntrophic relationships with other microorganisms that could shuttle the electron released by CH_4_, could explain gammaproteobacterial MOB activity in the absence of O_2_ in freshwater habitats (Fig. 3e).

#### O_2_ sources

Contrary to hypotheses of death and dormancy of MOB cells, activity-based evidence for CH_4_ oxidation in incubations with microoxic and anoxic lake water or sediment in the presence of proteobacterial methanotrophs suggests their viability and activity under O_2_-limited conditions (Fig. S1, Table S2). Despite apparently anoxic conditions, O_2_ supply to anoxic lake layers could support CH_4_ oxidation through episodic intrusions due to water column instability [30], downward diffusion from the upper mixed layer [72], or cryptic O_2_ cycling via local photosynthetic production [15, 17, 23, 73-75] (Fig. 3c and d). In bottle experiments, enhancement of CH_4_ oxidation by O_2_ amendment or light was observed in all known studies that tested for this potential (Fig. S1, Table S2). Milucka *et al*. [15] were the first to report a potential role for photosynthetic O_2_ production in sustaining CH_4_ oxidation below the oxycline. They showed that light treatments resulted in greater CH_4_ consumption compared with dark controls or controls spiked with an inhibitor of oxygenic photosynthesis and that no electron acceptor other than O_2_ increased CH_4_ oxidation in bottle incubations. These results indicate that CH_4_ oxidation coupled with oxygenic photosynthesis may be the dominant sink of CH_4_ in lakes where light penetrates below the chemocline, supporting the notion that O_2_ production by oxygenic phototrophs is tightly coupled to O_2_ consumption by aerobic processes under apparent anoxic conditions in aquatic ecosystems [76]. Following this study, others reported that both light exposure and/or O_2_ addition stimulated CH_4_ oxidation below the oxic-anoxic transition zone [17, 22, 23], including by the filamentous gammaproteobacterial *Crenothrix* in two Swiss lakes [73]. In anoxic lake sediments, an experimental treatment that received O_2_ addition showed CH_4_ oxidation 10-fold higher than controls or treatments with nitrite/nitrate additions [77]. Similarly, in a *Methylobacter*-dominated enrichment culture from a stratified lake, CH_4_ oxidation rates were two orders of magnitude higher under O_2_ saturation than under microoxic conditions, and no CH_4_ oxidation was observed under trace O_2_ or anoxic conditions [75].

Despite evidence from bottle experiments for cryptic O_2_ cycling in seemingly anoxic conditions of lakes, generation of the amount of O_2_ required to sustain fully aerobic methanotrophic metabolism according to theoretical stoichiometry (1CH_4_:2O_2_, Table S3; Fig. 1) may not be feasible *in situ*. For example, in Siberian lakes, the amount of O_2_ needed to support the rate of CH_4_ oxidation in the hypolimnion could not be explained by downward O_2_-diffusive flux or at-depth O_2_ production [55]. In these lakes, CH_4_ oxidation rates derived from a diffusion–reaction model applied to CH_4_ concentration profiles would require much higher O_2_ concentrations in overlaying waters or unreasonably high photosynthetic rates in the hypolimnion or on hypolimnetic sediments to sustain O_2_ consumption by CH_4_ oxidation. Such modeling data suggest that O_2_ supply alone cannot fully explain MOB activity under seemingly anoxic conditions in all lakes [55]. To overcome O_2_ supply limitation, it is possible that MOB efficiently use O_2_ in the MMO-mediated step of CH_4_ oxidation and support their metabolism by fermentation and/or anaerobic respiration using other electron acceptors (Fig. 1).

#### Alternative electron acceptors

Multiple alternative electron acceptors, other than O_2_, have been tested for their involvement in the metabolism of methanotrophic bacteria. In incubation experiments, additions of sulfate, nitrate, nitrite, iron oxides, manganese oxides, and humic substances all provided some evidence for stimulation of CH_4_ oxidation in anoxic sediments or waters where gammaproteobacterial MOB were the most abundant methanotrophs detected and anaerobic methanotrophic archaea were absent or negligible (see summary in Fig. S1, Table S2). Many other compounds found in freshwater ecosystems, such as quinones, dimethyl sulfoxide (DMSO), or trimethylamine N-oxide (TMAO), could also potentially be involved in the oxidation of CH_4_ in the absence of O_2_, provided that the oxygen-containing molecule is acceptable to the pMMO or sMMO enzymes or can function as an electron acceptor for anaerobic respiration. The oxidation of CH_4_ coupled to the reduction of different compounds is thermodynamically viable (e.g. Table S3), but the capability of MOB to use most of those alternative electron acceptors has not been demonstrated. Moreover, a metabolic pathway of CH_4_ oxidation completely independent of an external O_2_ source for activation of CH_4_ has not yet been identified for proteobacterial MOB representatives (Fig. 1). In addition, it remains unclear whether potential electron acceptors in incubations are involved in the activation of CH_4_ in the MMO-mediated step, are terminal electron acceptors in anaerobic respiration, are being used indirectly to build biomass (e.g. inorganic nitrogen sources, trace metals), or are favoring other microorganisms that are involved in consortia with methanotrophs. For example, despite stimulation of CH_4_ oxidation by the addition of alternative electron acceptors like metal oxides, often the amount of electron acceptors added in experiments or present *in situ* cannot account for the total CH_4_ oxidized based on the stoichiometry of the reactions (e.g. [16, 78], Table S3). Rissanen *et al*. [79] detected stimulation of CH_4_ oxidation by nitrate in boreal lake sediment containing multiple methanotrophs (namely gammaproteobacterial MOB, methanotrophic archaea (“Ca. Methanoperedens”), and “Ca. Methylomirabilota” MOB), and thus could not resolve a direct link between CH_4_ oxidation and the reduction of nitrate by gammaproteobacterial MOB specifically. Similarly, despite increased CH_4_ oxidation in incubations amended with nitrate and sulfate for Lacamas Lake (USA) samples, complete denitrification and sulfate reduction pathways were not detected in the metagenome-assembled genome (MAG) of the most abundant MOB population [67]. In iron-rich anoxic lake sediments, utilization of ferric oxides by MOB in a partnership with iron-reducing bacteria was proposed to mediate CH_4_ oxidation [80]. Riboflavin secreted by iron reducers was hypothesized to help transport electrons from CH_4_ to Fe^3+^ extracellularly, while iron-reducing bacteria assimilated CH_4_-derived carbon released by MOB. Likewise, labeling of DNA associated with *Methylobacter* (gammaproteobacterial MOB) and iron-reducing bacteria in ^13^CH_4_-based stable isotope probing experiments on sub-Arctic lake sediments [78] suggests that iron reducers assimilate organic compounds released by MOB under anoxia.

### Genetic makeup of aerobic methanotrophs under O_2_-limiting conditions

CH_4_ oxidation via MMO enzymes in methanotrophic bacteria is O_2_-dependent and can be coupled to aerobic respiration [44], anaerobic respiration (nitrate reduction [24]), or fermentation [25] (Fig. 1). Such metabolic versatility seems to allow MOB, particularly members of the order *Methylococcales*, to cope with O_2_ limitation in CH_4_-rich environments, such as bottom lake sediments and waters. Recent genomic studies have detected the presence of genes encoding fermentation and anaerobic respiration in *Methylococcales* genomes or MAGs recovered from anoxic freshwater ecosystems (Table 1; references therein). Similarly, to sustain aerobic CH_4_ oxidation under microoxic conditions, some *Methylococcales* populations appear to possess the genetic potential for high affinity oxidases, the O_2_ carrier hemerythrin, and extracellular electron transfer (Table 1).

**Table 1:** Genetic potential of gammaproteobacterial methanotrophs (gamma-MOB) detected in hypoxic or anoxic freshwater environments that could be related to their survival and activity under O_2_-limiting conditions.

Function	Genes	Taxa	Habitat	Reference
Fermentation	*sfcA*, *mdh*, *fumC*, *sdhABCD*, *pdhAB*, *ackA*, *pta*, *hoxFGHY*	*Methylococcales*, including *Methylobacter*	Anoxic lake water	[64, 67]
Cytochrome *c* and *bd* oxidases (high-affinity oxidases)	*cydA*, *cox1*	*Methylococcales*	Lake water, wetland	[26, 64, 67]
Bacteriohemerythrin (O_2_ carrier)	McHr	*Methylococcus*	Lake water	[64]
Dissimilatory nitrate reduction	*narGHIJ*/*napAB*	*Methylococcales*, including *Methylobacter* and *Crenothrix*	Lake sediment and water, wetland	[17, 64, 81, 82]
Nitrite reduction	*nirK*/*nirS*/*aniA*	*Methylococcales*, including *Methylobacter* and *Crenothrix*	Lake sediment and water, wetland	[17, 64, 75, 81, 82]
Nitric oxide reduction	*norBC*	*Methylococcales*	Lake water, wetland	[64, 82]
Iron reduction/oxidation (extracellular electron transfer)	*mtoA*, *mtrB*	*Methylococcales*	Lake water	[64]
Electrically conductive pili (extracellular electron transfer)	*pilA*	*Methylomonas*	Anoxic lake sediment	[80]
Electron shuttle riboflavin (extracellular electron transfer)	*ribA*, *ribBA*, *ribD*, *ribE*, *ribF*, *ribH*	*Methylomonas*	Anoxic lake sediment	[80]

Terminal oxidases are the enzymes responsible for complete reduction of O_2_ to water during aerobic respiration. These enzymes have a range of affinities for O_2_ in bacteria. Microorganisms possessing high-affinity oxidases are assumed to have the ability to perform aerobic respiration at low O_2_ concentrations, which can be advantageous where O_2_ availability is spatially and temporally dynamic. Genes encoding high-affinity cytochrome *c* and *bd* oxidases have been detected in members of *Methylococcales* and may facilitate aerobic respiration under trace O_2_ concentrations of lakes and wetlands [26, 64].

Additionally, aerobic methanotrophs possess non-heme iron containing proteins called hemerythrin (Hr) or bacteriohemerythrin (Bhr), which are O_2_ carriers thought to deliver cytosolic O_2_ to the membrane-bound pMMO for CH_4_ oxidation [27, 28]. Indeed, efficient activity of over-expressed pMMO in *Methylococcus capsulatus* Bath was suggested to be affected by Bhr delivery of dioxygen from the cytoplasm to intra-cytoplasmatic membranes [27]; and upregulation of Bhr genes has been observed in methanotrophs under O_2_ limitation [24, 25]. Others reported that Bhr overproduction in methanotrophic strains led to increased O_2_ consumption, but minimal O_2_ input to CH_4_ oxidation machinery, and suggested that Bhr proteins specifically contribute to aerobic respiration rather than aerobic CH_4_ oxidation [28]. Regardless, gammaproteobacterial methanotroph genomes were shown to prominently encode either Hr or Bhr protein domains [83], supporting that the presence of these proteins could be a widespread strategy of methanotrophs to improve the respiration or CH_4_ oxidation efficiency when O_2_ supply is limiting.

The presence of gas vesicles within methanotrophs may also help influence their distribution and activity in dynamic and O_2_-scarce environments. Gas vesicles are protein-encased cylinders that are permeable to gas and provide buoyancy to aquatic microorganisms [84]. In aerobic methanotrophs inhabiting microoxic or anoxic niches, gas vesicles could hypothetically provide buoyancy for adjusting their position in the water column as well as increase the contact surface inside the cell optimizing O_2_ usage under low O_2_ conditions. Within canonical aerobic methanotrophs, a psychrophilic *Methylococcus*-like strain (*Gammaproteobacteria*) isolated from tundra soil contained gas vesicles [85]. The species *Methylosphaera hansonii* (order *Methylococcales*, *Gammaproteobacteria*), isolated from the hypolimnion and benthic zones of an Antarctic meromictic lake, was also reported to contain gas vesicles [86]. Within bacterial methanotrophs in the “Ca. Methylomirabilota” phylum, a gene cluster encoding several gas vesicle-related proteins (*gvpA*, *gvpL/F*, *gvpN*, and *gvpK*) was found to be well transcribed during a bloom of “*Ca.* Methylomirabilis limnetica” in Lake Zug, Switzerland [87]. Gas vesicle genes or proteins have also been detected in methanotrophic archaeal MAGs or proteomes. A gene cluster for gas vesicle production, *gvpF*-*gvpO*-*gvpN*-*gvpA*, was observed in a MAG affiliated with Ca. *Methanoperedens psychrophilus*, and it was hypothesized that the gas vesicles may serve to store CH_4_ [88]. Similarly, in marine cold seep sediments, the proteome of ANME-1 included a gas vesicle synthesis family protein and gas vesicle protein GvpN, although the physiological function of these proteins remains uncertain [89].

In cultures, the gammaproteobacterial methanotroph *Methylomicrobium alcaliphilum* strain 20Z was found to couple CH_4_ utilization to fermentation, with formate, acetate, succinate, lactate, and hydroxybutyrate as end products, which are excreted [25] (Fig. 1). Genomic analyses have also revealed fermentation pathways in gammaproteobacterial MOB representatives detected in lakes. In the stratified temperate Lacamas Lake (USA), *Methylobacter* MAGs retrieved from the anoxic hypolimnion encoded a fermentation pathway, besides other adaptations to O_2_ limitation [67]. Similarly, in a boreal Finnish lake, several *Methylococcales* MAGs possessed genes encoding the fermentation pathway [64] (Table 1).

Using isolates and transcriptome data, Kits *et al*. [24] showed that, under low O_2_ concentration and nitrate availability, *Methylomonas denitrificans* couples CH_4_ oxidation to incomplete nitrate reduction, releasing nitrous oxide as a terminal product (Fig. 1). Their results suggested that the genetic makeup encoding the denitrification pathway in *M. denitrificans* is upregulated only under O_2_ limitation, indicating a strategy to cope with low O_2_ availability. Genomic surveys of freshwater ecosystems have detected genomic potential for partial denitrification pathways in multiple *Methylococcales*-associated MAGs (Table 1).

Methanotrophic bacteria may also possess the capacity for extracellular electron transfer. A recent study proposed that *Methylococcales* members may couple CH_4_ oxidation with extracellular electron transfer to iron oxides under 2% O_2_ conditions [80]. The addition of ferric iron (Fe^3+^) to a 2% O_2_ treatment enhanced CH_4_ oxidation by 40%, and dissolved Fe^2+^ concentrations increased, indicating that iron oxides serve as electron acceptors for MOB when O_2_ availability is low [80]. Riboflavin, an electron shuttle that is known to be produced by some bacterial species [90], was present at higher concentrations in the 2% O_2_ treatment, suggesting that riboflavin is a potential mediator for iron reduction by MOB under anoxia. Accordingly, genes encoding riboflavin and electrically conductive pili (e-pili), both potentially involved in the extracellular electron transfer, were present in *Methylomonas*-associated MAGs derived from anoxic lake sediment samples [80].

### Future perspectives

The study of CH_4_ oxidation by aerobic methanotrophic bacteria under O_2_-limited conditions is an area of active research with many possibilities for future investigation. Here we highlight several research directions that could help improve our understanding of the persistence and activity of these aerobic microorganisms under O_2_ deficiency in freshwaters.

Future research should test CH_4_ oxidation by MOB under zero O_2_ conditions and in the presence of *in situ* concentrations of alternative electron acceptors. To accomplish this, there is a need for developing methods and practices for avoiding O_2_ contamination during sample collection and manipulation. Those include sampling methods that minimize gas exchange, such as extensive overflow of sampling bottles, or use of tubing directly connected to N_2_/He filled bottles, or filling of experimental bottles at depth. In the laboratory, lake water or culture medium should be flushed with N_2_/He and an anaerobic chamber should be used for sample manipulation during experimental setup. Experimental vessels and stoppers should be gas-tight and bottles slightly over pressurized to avoid O_2_ intrusion from ambient air. Also, the application of methanogenic inhibitors [e.g. bromoethane sulfonate (BES)] in incubation experiments with lake water or sediment could potentially provide important insights. This inhibitor acts on the MCR enzyme, thereby inhibiting the growth and activity of anaerobic CH_4_-producing and oxidizing archaea [91, 92]. Using BES or other inhibitors of archaea in laboratory experiments with anoxic lake water or sediment, it may be possible to detect CH_4_ oxidation by aerobic methanotrophic bacteria only. The use of stable isotopes to track CH_4_ oxidation under anoxia is another powerful approach and can be particularly useful in this context when methanogenesis is interrupted using an inhibitor. Measuring carbon and/or hydrogen isotopic compositions for CH_4_ in anoxic incubations where only bacterial oxidation is occurring will provide critical knowledge on the isotopic fractionation of such a process and facilitate whole-ecosystem inferences of anaerobic CH_4_ oxidation by aerobic MOB using stable isotope mass balances (e.g. [6]). Additionally, stable-isotope probing can be used to track the fate of specific carbon sources in microbial communities (e.g. [93]). By labeling CH_4_ with a stable isotope such as ^13^C and tracking its incorporation into microbial biomass or other products through techniques such as fluorescence *in situ* hybridization coupled to nanoscale secondary ion mass spectrometry (FISH-nanoSIMS) or DNA/RNA sequencing of the heavy and light fractions, researchers have gained and can continue to derive new insight into the identity of the microbial players and the genes involved in the oxidation of CH_4_ under seemingly anoxic conditions (e.g. [15, 16, 68, 78]).

Exploration of syntrophic interactions potentially involved in anaerobic CH_4_ oxidation by aerobic methanotrophs should involve testing microbial interactions that occur in anoxic environments where aerobic methanotrophic bacteria appear to be active and oxidizing CH_4_. For example, conducting methanotrophic enrichment cultures under specific conditions (e.g. light, amended electron acceptors) could potentially enrich microbial consortia involved in the oxidation of CH_4_, such as MOB and oxygenic photosynthesizers, MOB and bacteria that use alternative electron acceptors (e.g. sulfate-reducing bacteria), or MOB and bacteria that produce oxidants that could be used by MOB during CH_4_ oxidation (e.g. photoferrotrophs producing Fe^3+^, which could potentially be used as electron acceptor for respiration coupled to CH_4_ oxidation). Understanding such interactions would enlighten the potential role of microbial partnerships in the oxidation of CH_4_ in O_2_-limited environments and the role of alternative electron acceptors in the metabolism of aerobic methanotrophs.

Isolation and characterization of methanotrophic strains would better delineate phylogenetic and functional diversity of methanotrophic bacteria with respect to their capabilities under different environmental conditions. Future research could focus on isolating and characterizing lake-associated methanotrophic strains that are capable of thriving under microoxic and anoxic conditions (e.g. [94]). This would enable, for instance, investigation of the role of bacteriohemerythrin (Bhr), gas vesicles, high-affinity oxidases, and extracellular electron transfer in the microbial oxidation of CH_4_ under varying O_2_ conditions. This could also involve screening environmental samples for methanotrophs combined with high-throughput techniques, such as metagenomics and metatranscriptomics, to identify their metabolic pathways and characteristics. Studying the physiology, metabolism, and genetic makeup of these bacteria will help elucidate the mechanisms enabling CH_4_ oxidation under O_2_-limiting conditions.

Given the importance of methanotrophy for global climate and biogeochemical cycling [1], and the ecology of limnetic systems [65], future research should continue to evaluate the prevalence and significance of aerobic CH_4_ oxidation under seemingly anoxic conditions in stratified lakes, as well as gain a deeper understanding of the mechanisms and limitations of microbial consumption of this potent greenhouse gas. This could alter the way aquatic scientists and microbial ecologists currently understand, model, and predict the CH_4_ cycle in freshwaters. For instance, if the link between CH_4_ oxidation and photosynthetic O_2_ production is widespread in lakes, then changes in the underwater light regime due to, e.g. eutrophication or lake water browning, will have direct impacts on the CH_4_ consumption in deep waters and sediments. Such knowledge is particularly urgent given predictions of future increased CH_4_ production and emissions in warmer and eutrophic lake ecosystems [13, 14].

## Supplementary Material

Reisms_supp_wrae041

## Data Availability

There are no primary data associated with this review article.
