# Supplementary material for: Enigmatic persistence of aerobic methanotrophs in oxygen-limiting freshwater habitats
Source: ISME J. 2024 Mar 12;18(1):wrae041. doi: 10.1093/ismejo/wrae041 (PMC11008690; doi:10.1093/ismejo/wrae041)
Supplement: Reisms_supp_wrae041 [file reisms_supp_wrae041.docx]

Supplemental Material

**Enigmatic persistence of aerobic methanotrophs in oxygen-limiting freshwater habitats**

Paula C. J. Reis, Jackson M. Tsuji, Cerrise Weiblen, Sherry L. Schiff, Matthew Scott, Lisa Y. Stein, Josh D. Neufeld

Content:

Figure S1………….…………………………………………………………………………page 2

Table S1.………….…………………………………………………………………………page 3

Table S2...……….………………………………...…………………………………………page 4

Table S3..………….…………………………………………………………………………page 8

References..………….………………………………………………………………………page 9

**
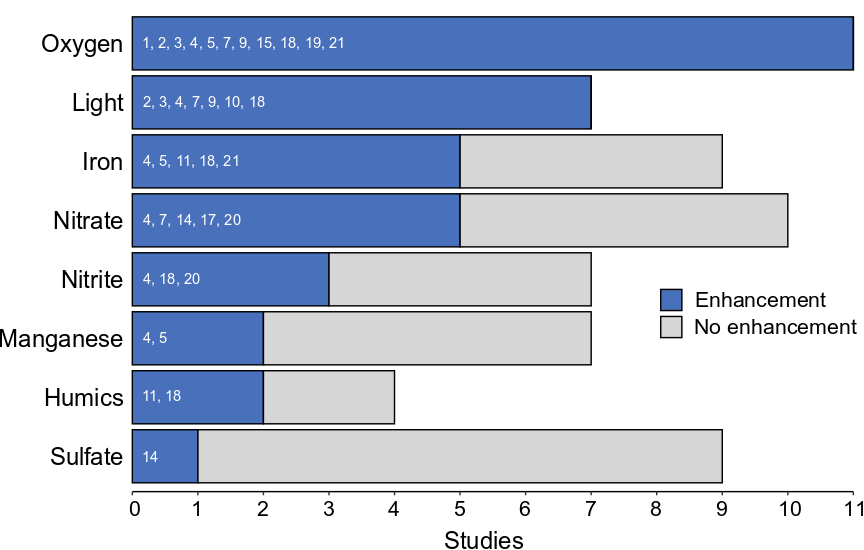
Figure S1**. Effect of the addition of electron acceptors and exposure to light on methane oxidation rate by methane-oxidizing bacteria in anoxic freshwater environments. Depicted are the results of known studies that tested at least one electron acceptor or condition in incubation experiments (subset of references in Table S2). Enhancement was considered as the increase in methane oxidation (relative to a control) by the addition of an electron acceptor or by light in at least one sampling site, lake, or depth reported by a study. Numbers within bars indicate the references that showed enhancement of methane oxidation under the condition tested.

**Table S1**. Taxonomic distribution of known aerobic and anaerobic methanotrophic microorganisms.

| Pathway of methane oxidation | Domain | Phylum/Class | Order/Family/Clade | Genera |
| --- | --- | --- | --- | --- |
| Aerobic  (methane monooxygenase-MMO enzyme) | Bacteria  (methane-oxidizing bacteria-MOB) | *Gammaproteobacteria* | *Methylococcales* | all |
|  |  | *Alphaproteobacteria* | *Methylocystaceae* | *Methylocystis*  *Methylosinus* |
|  |  |  | *Beijerinckiaceae* | *Methylocella Methylocapsa Methyloferula* |
|  |  | *Verrucomicrobia* |  | *Methylacidiphilum*  *Methylacidimicrobium* |
|  |  | “*Ca*. Methylomirabilota” (formerly NC10) | *Methylomirabilales* | “*Ca.* Methylomirabilis oxyfera” |
| Anaerobic  (methyl coenzyme M reductase-MCR enzyme) | Archaea  (Anaerobic methanotrophic archaea-ANME) | *Euryarchaeota*/  *Methanomicrobia* | *Methanophagales* (formely ANME-1) |  |
|  |  |  | *Methanosarcinales*/  *Methanocomedenaceae* (ANME-2a/b) |  |
|  |  |  | *Methanosarcinales*/  *Methanogasteraceae* (ANME-2c) |  |
|  |  |  | *Methanosarcinales*/  *Methanoperedenaceae* (formerly ANME-2d) | “*Ca.* Methanoperedens” |
|  |  |  | *Methanosarcinales*/  *Methanosarcinaceae* | “*Ca.* Methanovorans” (ANME-3) |

| **Table S2**. Summary of studies that reported methane oxidation rates by aerobic methanotrophic bacteria under O_2_-limiting conditions in lakes. | | | | | | |
| --- | --- | --- | --- | --- | --- | --- |
| Lake | Habitat | Determination of methane oxidation rate | Tested electron acceptors/conditions | Electron acceptors/conditions that enhanced methane oxidation | Microorganisms involved (method of identification or quantification) | Reference |
| Lugano (North basin; Switzerland) | Water column | Radioactivity (^14^C) of residual CH_4_ and produced CO_2_ in incubations | O_2_ | O_2_ | *Methylobacter* (gamma-MOB)  (PLFA, *pmo*A gene sequencing) | [1] |
| di Cadagno (Switzerland) | Water column | ^13^CO_2_ production in incubations | O_2_, light, DCMU, iron, manganese, nitrite, sulfate | O_2_, light | gamma-MOB  (CARD-FISH, nanoSIMS, *pmo*A gene sequencing) | [2] |
| Rotsee (Switzerland) | Water column | ^13^CO_2_ production in incubations | O_2_, light, DCMU | O_2_, light | gamma-MOB  (CARD-FISH, nanoSIMS) | [3] |
| La Cruz (Spain) | Water column | ^13^CO_2_ production in incubations | O_2_, light, nitrate, nitrite, iron (ferrihydrate) and manganese (birnessite) oxides | O_2_, light, and slight stimulation by nitrate, nitrite, iron, and manganese | Aerobic methanotrophic bacteria  (CARD-FISH, 16S rRNA gene and *pmo*A gene sequencing) | [4] |
| Zug (Switzerland) | Water column | ^13^CO_2_ production in incubations | O_2_, nitrate, nitrite, Fe^3+^, Mn^4+^, sulfate | O_2_; Fe^3+^ and Mn^4+^ but inconclusive (the added amount could not account for the amount ^13^CO_2_ produced) | gamma-MOB  (CARD-FISH, nanoSIMS) | [5] |
| Vault (Alaska, USA) | Sediment | Decrease in ^13^CH_4_ concentration in incubations | None (no amendments) | NA | *Methylobacter* (gamma-MOB)  (DNA- and PLFA-SIP) | [6] |
| Rotsee and Zug (Switzerland) | Water column | ^13^CO_2_ production in incubations | O_2_, light, nitrate | O_2,_ light, nitrate | *Crenothrix* (gamma-MOB)  (CARD-FISH, nanoSIMS, 16S rRNA gene sequencing, metagenomics) | [7] |
| Doughnut and Vault (Alaska), Dagow (Germany) | Sediment | Decrease in ^13^CH_4_ concentration in incubations | None (no amendments) | NA | Aerobic methanotrophic bacteria  (qPCR of *pmo*A gene) | [8] |
| Alinen-Mustajärvi and Mekkojärvi (Finland) | Water column | ^13^C-DIC production in incubations | O_2_, light, sulfate, nitrate, Fe^3+^, Mn^4+^, anthraquinone-2,6-disulfonate | O_2_ and light | gamma-MOB (*Methylobacter* and Ca. *Methyloumidiphilus alinensis*)  (DNA- and RNA-based amplicon sequencing, shotgun metagenomics) | [9] |
| Lake Svetloe (Russia) | Water column and sediment | Radioactivity of residual CH_4_ and decrease in CH_4_ concentration over time | Light, DCMU, Fe^3+^ | Light | *Methylobacter* and other unknown potential partners (16S rRNA gene sequencing) | [10] |
| NA (*Methylomona*s and *Methylosinus* strains) | NA | CO_2_ production in incubations | Fe^3+^ (ferrihydrite), ADQS | Fe^3+^ (ferrihydrite), ADQS | *Methylomonas* (gamma-MOB) and *Methylocystis* (alpha-MOB)  (16S rRNA gene sequencing) | [11] |
| L1, L2, L3, and L4 (Siberia) | Water column | Diffusion-reaction model applied to CH_4_ concentration profile | NA | NA | *Methylobacter* and other *Methylomonadaceae* (gamma-MOB)  (16S rRNA gene sequencing, qPCR of *pmo*A gene) | [12] |
| Sila (Siberia) | Water column | Diffusion-reaction model applied to CH_4_ concentration profile | NA | NA | Aerobic methanotrophic bacteria  (qPCR of *pmo*A gene) | [13] |
| Lacamas (USA) | Water column | Decrease in CH_4_ concentration in incubations | O_2_, nitrate, sulfate | Nitrate, sulfate | *Methylobacter* clade 2 (gamma-MOB)  (16s rRNA and *pmo*A genes sequencing, MAG) | [14] |
| Lacamas (USA) | Water column (enrichment culture) | Decrease in CH_4_ concentration in incubations | O_2_, nitrate, sulfate, humic substances | O_2_ saturation | *Methylobacter* (gamma-MOB)  (16S rRNA gene sequencing, qPCR of 16S rRNA gene, MAG) | [15] |
| Qalluuraq (Alaska, USA) | Sediment | Decrease in CH_4_ concentration in incubations | Sulfate | None | gamma-MOB (*Methylobacter* being the most abundant)  (DNA-SIP) | [16] |
| Pääjärvi (Finland) | Sediment | ^13^C-DIC production in incubations | Nitrate | Nitrate (in only one sampled station) | Methanotrophic archaea (Ca. *Methanoperedens*), potentially *Methylococcales* (gamma-MOB) and *Methylomirabilis* (*Ca*. Methylomirabilota, formerly NC10)  (16S rRNA gene sequencing, shotgun metagenomics) | [17] |
| Lovojärvi (Finland) | Water column | ^13^C-DIC production in incubations | O_2_, light, nitrate, nitrite, Fe^3+^, Mn^4+^, ADQS, humic acids | O_2_, light, nitrite, Fe^3+^, humic acids (at different depths) | *Methylobacter*, *Crenothrix*, *Methyloparacoccus* (gamma-MOB)  (CARD-FISH, 16S rRNA gene sequencing) | [18] |
| Sempach (Switzerland) | Sediment | Radioactivity (^14^C) of residual CH_4_ and produced CO_2_ in incubations; change in ^13^C-DIC with incubation time | O_2_, nitrate, nitrite, sulfate, iron, and manganese oxides | O_2_ | *Methylobacter* (gamma-MOB), *Methylocystis* (alpha-MOB), and potentially *Crenothrix* (gamma-MOB)  (16S rRNA gene sequencing, ^13^C lipid probing) | [19] |
| Lugano (North and South basin; Switzerland) | Water column | Radioactivity (^14^C) of residual CH_4_ and produced CO_2_ in incubations | Nitrate, nitrite, sulfate | None (South basin); nitrate and nitrite (North basin) | *Methylobacter*, *Crenothrix* (South basin); *Methylobacte*r, *Crenothrix* and Ca. *Methylomirabilis* (North basin)  (16S rRNA gene sequencing) | [20] |
| Fuxian (China) | Sediment | ^13^CO_2_ production in incubations | O_2_, nitrate, nitrite, Mn^4+^ and Fe^3+^ oxides, sulfate | Fe^3+^ in the 2% O_2_ treatment only | *Methylomonas* (gamma-MOB)  (DNA-SIP) | [21] |

NA: does not apply. CARD-FISH: catalyzed reporter deposition fluorescence *in situ* hybridization. nanoSIMS: nanometer-scale secondary ion mass spectrometry. SIP: stable isotope probing. MAG: metagenome-assembled genome. DIC: dissolved inorganic carbon. qPCR: quantitative polymerase chain reaction.

**Table S3**. Examples of potential electron acceptors in methane oxidation (thermodynamically feasible, i.e., ∆G°’ < 0) with respective theoretical stoichiometry and reported Gibbs free energy changes.

| Electron acceptor | Reaction | ∆G°’ (kJ mol^-1^ CH_4_) | Reference |
| --- | --- | --- | --- |
| Oxygen | CH_4_ + 2O_2_ $\to$ CO_2_ + 2H_2_O | -858 | [22] |
| Nitrite | 3CH_4_ + 8NO_2_^-^ + 8H^+^ $\to$ 3CO_2_ + 4N_2_ + 10H_2_O | -928 | [23, 24] |
| Nitrate | 5CH_4_ + 8NO_3_^-^ + 8H^+^ $\to$ 5CO_2_ + 4N_2_ + 14H_2_O | -765 to -801 | [23, 25] |
| Manganese (Mn^4+^) | CH_4_ + 4MnO_2_ + 7H^+^ $\to$ HCO_3_^-^ + 4Mn^2+^ + 5H_2_O | -790 | [25] |
| Iron (Fe^3+^) | CH_4_ + 8Fe(OH)_3_ + 15H^+^ $\to$ HCO_3_^-^ + 8Fe^2+^ + 21H_2_O | -571 | [25] |
| AQDS* | CH_4_ + 4ADQS + 3H_2_O $\to$ HCO_3_^-^ + H^+^ + 4AQH_2_DS | -41 | [26] |
| Sulfate | CH_4_ + SO_4_^2-^ + 2H^+^ $\to$ CO_2_ + H_2_S + 2H_2_O | -16 | [27] |

* 9,10-anthraquinone-2,6-disulfonate (humic compound)

**References**

1. Blees J, Niemann H, Wenk CB, Zopfi J, Schubert CJ, Kirf MK, et al. Micro-aerobic bacterial methane oxidation in the chemocline and anoxic water column of deep south-Alpine Lake Lugano (Switzerland). *Limnol Oceanogr* 2014; **59**: 311–324.

2. Milucka J, Kirf MK, Lu L, Krupke A, Lam P, Littmann S, et al. Methane oxidation coupled to oxygenic photosynthesis in anoxic waters. *ISME J* 2015; **9**: 1991–2002.

3. Oswald K, Milucka J, Brand A, Littmann S, Wehrli B, Kuypers MMM, et al. Light-dependent aerobic methane oxidation reduces methane emissions from seasonally stratified lakes. *PLoS One* 2015; **10**: e0132574.

4. Oswald K, Jegge C, Tischer J, Berg J, Brand A, Miracle MR, et al. Methanotrophy under versatile conditions in the water column of the ferruginous meromictic Lake La Cruz (Spain). *Front Microbiol* 2016; **7**: 1762.

5. Oswald K, Milucka J, Brand A, Hach P, Littmann S, Wehrli B, et al. Aerobic gammaproteobacterial methanotrophs mitigate methane emissions from oxic and anoxic lake waters. *Limnol Oceanogr* 2016; **61**: S101–S118.

6. Martinez-Cruz K, Leewis M-C, Herriott IC, Sepulveda-Jauregui A, Anthony KW, Thalasso F, et al. Anaerobic oxidation of methane by aerobic methanotrophs in sub-Arctic lake sediments. *Sci Total Environ* 2017; **607**–**608**: 23–31.

7. Oswald K, Graf JS, Littmann S, Tienken D, Brand A, Wehrli B, et al. *Crenothrix* are major methane consumers in stratified lakes. *ISME J* 2017; **11**: 2124–2140.

8. Martinez-Cruz K, Sepulveda-Jauregui A, Casper P, Anthony KW, Smemo KA, Thalasso F. Ubiquitous and significant anaerobic oxidation of methane in freshwater lake sediments. *Water Res* 2018; **144**: 332–340.

9. Rissanen AJ, Saarenheimo J, Tiirola M, Peura S, Aalto SL, Karvinen A, et al. Gammaproteobacterial methanotrophs dominate methanotrophy in aerobic and anaerobic layers of boreal lake waters. *Aquat Microb Ecol* 2018; **81**: 257–276.

10. Kallistova A, Kadnikov V, Rusanov I, Kokryatskaya N, Beletsky A, Mardanov A, et al. Microbial communities involved in aerobic and anaerobic methane cycling in a meromictic ferruginous subarctic lake. *Aquat Microb Ecol* 2018; **82**: 1–18.

11. Zheng Y, Wang H, Liu Y, Zhu B, Li J, Yang Y, et al. Methane-dependent mineral reduction by aerobic methanotrophs under hypoxia. *Environ Sci Technol Lett* 2020; **7**: 606–612.

12. Cabrol L, Thalasso F, Gandois L, Sepulveda-Jauregui A, Martinez-Cruz K, Teisserenc R, et al. Anaerobic oxidation of methane and associated microbiome in anoxic water of Northwestern Siberian lakes. *Sci Total Environ* 2020; **736**: 139588.

13. Thalasso F, Sepulveda-jauregui A, Gandois L, Martinez-cruz K, Gerardo-nieto O, María SA, et al. Sub-oxycline methane oxidation can fully uptake CH4 produced in sediments: case study of a lake in Siberia. 2020; **10**: 3423.

14. van Grinsven S, Sinninghe Damsté JS, Abdala Asbun A, Engelmann JC, Harrison J, Villanueva L. Methane oxidation in anoxic lake water stimulated by nitrate and sulfate addition. *Environ Microbiol* 2020; **22**: 766–782.

15. van Grinsven S, Sinninghe Damsté JS, Harrison J, Villanueva L. Impact of electron acceptor availability on methane-influenced microorganisms in an enrichment culture obtained from a stratified lake. *Front Microbiol* 2020; **11**: 715.

16. He R, Wang J, Pohlman JW, Jia Z, Chu YX, Wooller MJ, et al. Metabolic flexibility of aerobic methanotrophs under anoxic conditions in Arctic lake sediments. *ISME J* 2022; **16**: 78–90.

17. Rissanen AJ, Jilbert T, Simojoki A, Mangayil R, Aalto SL, Peura S, et al. Anaerobic oxidation of methane in sediments of a nitrate-rich, oligo-mesotrophic boreal lake. *bioRxiv* 2021; 2021.02.12.426818.

18. van Grinsven S, Oswald K, Wehrli B, Jegge C, Zopfi J, Lehmann MF, et al. Methane oxidation in the waters of a humic-rich boreal lake stimulated by photosynthesis, nitrite, Fe(III) and humics. *Biogeosciences* 2021; **18**: 3087–3101.

19. Su G, Zopfi J, Niemann H, Lehmann MF. Multiple groups of methanotrophic bacteria mediate methane oxidation in anoxic lake sediments. *Front Microbiol* 2022; **13**: 864630.

20. Su G, Lehmann MF, Tischer J, Weber Y, Lepori F, Walser JC, et al. Water column dynamics control nitrite-dependent anaerobic methane oxidation by *Candidatus* “Methylomirabilis” in stratified lake basins. *ISME J* 2023; **17**: 693–702.

21. Li B, Tao Y, Mao Z, Gu Q, Han Y, Hu B, et al. Iron oxides act as an alternative electron acceptor for aerobic methanotrophs in anoxic lake sediments. *Water Res* 2023; **234**: 119833.

22. Reed DC, Deemer BR, Grinsven S Van, Harrison JA. Are elusive anaerobic pathways key methane sinks in eutrophic lakes and reservoirs? *Biogeochemistry* 2017; **134**: 29–39.

23. Raghoebarsing AA, Pol A, Van De Pas-Schoonen KT, Smolders AJP, Ettwig KF, Rijpstra WIC, et al. A microbial consortium couples anaerobic methane oxidation to denitrification. *Nature* 2006; **440**: 918–921.

24. Ettwig KF, Butler MK, Le Paslier D, Pelletier E, Mangenot S, Kuypers MMM, et al. Nitrite-driven anaerobic methane oxidation by oxygenic bacteria. *Nature* 2010; **464**: 543–548.

25. Segarra KEA, Comerford C, Slaughter J, Joye SB. Impact of electron acceptor availability on the anaerobic oxidation of methane in coastal freshwater and brackish wetland sediments. *Geochim Cosmochim Acta* 2013; **115**: 15–30.

26. Scheller S, Yu H, Chadwick GL, McGlynn SE, Orphan VJ. Artificial electron acceptors decouple archaeal methane oxidation from sulfate reduction. *Science* 2016; **351**: 703–707.

27. Knittel K, Boetius A. Anaerobic oxidation of methane: progress with an unknown process. *Annu Rev Microbiol* 2009; **63**: 311–334.
